# The prognostic value and biological significance of gap junction beta protein 2 (GJB2 or Cx26) in cervical cancer

**DOI:** 10.3389/fonc.2022.907960

**Published:** 2022-07-21

**Authors:** Silu Meng, Yuhuan Liu, Xiaoyan Wang, Xue Wu, Wan Xie, Xiaoyan Kang, Xiaoyu Liu, Lili Guo, Changyu Wang

**Affiliations:** ^1^ Department of Obstetrics and Gynecology, Tongji Hospital, Tongji Medical College, Huazhong University of Science and Technology, Wuhan, China; ^2^ Cancer Biology Research Center, Tongji Hospital, Tongji Medical College, Huazhong University of Science and Technology, Wuhan, China; ^3^ Institute of Pathology, Tongji Hospital, Tongji Medical College, Huazhong University of Science and Technology, Wuhan, China

**Keywords:** GJB2, cervical cancer, prognostic marker, immune cell abundance, chemoresistance

## Abstract

**Objective:**

To evaluate the prognostic value and explore the biological significance of gap junction protein beta 2 (GJB2 or Cx26) in cervical cancer (CC).

**Methods:**

We first compared GJB2 expression between CC and normal tissues using public databases and immunohistochemistry (IHC). Based on The Cancer Genome Atlas data (TCGA cohort, n = 304) and tissue microarray samples (OBC cohort, n = 111), we explored the prognostic value of GJB2 for CC patients using bioinformatics analysis and IHC scoring. To explore the biological significance of GJB2, Gene set enrichment analysis (GSEA) and Gene Ontology (GO) were performed. The impact of GJB2 on the immune microenvironment was analyzed by CIBERSORTx and ESTIMATE algorithms. We finally investigated the relationship between GJB2 and drug sensitivity based on the Genomics of Drug Sensitivity in Cancer (GDSC).

**Results:**

The expression of GJB2 was significantly increased in CC over normal tissues. Both the TCGA and OBC cohort found that patients with high GJB2 expression had shorter overall survival (OS) time, and high GJB2 expression was the independent risk factor for prognosis (TCGA: HR, 2.566; 95% CI, 1.066–6.180; p = 0.036; OBC: HR, 2.198; 95% CI, 1.019–4.741; p = 0.045). GJB2 was correlated with patient clinical factors such as tumor size and differentiation grade. The p53 signaling pathway and toll-like receptor pathway may be regulated by GJB2. The abundance of various immune cells was significantly different between the low and high GJB2 expression groups. The ImmuneScore was significantly increased in the high GJB2 expression group. In addition, the expression level of GJB2 was positively correlated with the natural log of the half-maximal inhibitory concentration (LN_IC50) value of cisplatin/paclitaxel (Spearman r = 0.238/0.153, p < 0.001).

**Conclusion:**

GJB2 can serve as a potential prognostic marker of poor survival and a therapeutic target in CC. Moreover, GJB2 may affect the immune microenvironment and is correlated with chemoresistance.

## Introduction

Cervical cancer (CC) is the fourth most frequently diagnosed cancer and the fourth leading cause of cancer death in women, with an estimated 604 127 new cases and 341 831 deaths worldwide for 2020 (1). Although the incidence and mortality rates have declined for the past few decades due to the screening program and Human Papillomavirus (HPV) vaccine, the risk of this disease in younger women has increased in some countries, and pre-mature CC mortality rapidly increases in areas without effective screening measurements (1–5). The 5-year overall survival (OS) for CC varies among countries, ranging from 60–69 in 34 countries and below 60% in a further 20 countries (6). The recurrence rate for the Federation of Gynecology and Obstetrics (FIGO) stage IB-IIA and IIB-IVA patients are 11%–22% and 28%–64%, respectively. Furthermore, the 5-year OS rate for recurrent CC is less than 5% despite intensive therapy (7, 8).

The gap junction protein family, also known as the connexin family, plays a critical role in gap junction intercellular communication (GJIC) (9). Reduced expression or altered cytoplasmic localization of connexins results in loss of GJIC in tumor cells (9). Gap Junction Protein Beta 2 (GJB2), also known as connexin 26 (Cx26), is one of the most widely studied members of this family (9). GJB2 has been shown to be correlated with the prognosis of cancer patients. Naoi et al. reported that GJB2 expression was associated with lymphatic vessel invasion, large tumor size, high histological grade, and poor relapse-free survival (10). In a study of colorectal cancer, high GJB2 expression was related to venous invasion, lung metastasis, and poor disease-free survival (11). Overexpression of GJB2 has been identified to promote tumor growth, migration, and invasion *via* the PI3K/Akt pathway, and knockdown of GJB2 could reduce migration and invasion (12). In addition, Teleki et al. found that GJB2 expression decreased post-chemotherapy compared to pre-chemotherapy and was associated with better survival in breast cancer, suggesting that GJB2 expression correlated with the response to chemotherapy (13). Connexins have the potential to serve as targets to develop drugs against metastasis and chemoresistance, such as the metastasis inhibitor-18, which can inhibit GJB2-mediated GJIC (9, 14).

This study aimed to explore the prognostic significance of GJB2 and its function in CC. Based on The Cancer Genome Atlas (TCGA) and Gene Expression Omnibus (GEO) databases, we analyzed the relationship between GJB2 and clinical parameters and its prognostic value for CC. We further validated the predictive value of GJB2 on the prognosis using immunohistochemistry (IHC). Gene set enrichment analysis (GSEA), Gene Ontology (GO) analysis, immune cells abundance analysis, and chemoresistance analysis were performed to explore the function of GJB2.

## Methods

### GJB2 expression analysis

GEPIA (http://gepia.cancer-pku.cn/) was used to compare the mRNA expression levels of GJB2 in patients with CC (based on the TCGA database) and normal controls (based on the GTEx project) (15). The Human Protein Atlas (https://www.proteinatlas.org/) was also used to explore the expression of GJB2 at the protein level. We further compared the expression of GJB2 in the paired CC and paracancerous tissue by IHC.

### Data download and collation

The RNA-seq data (304 cases; Workflow Type: HTSeq - FPKM-UQ) and clinical information of CC were downloaded from the TCGA database (https://portal.gdc.cancer.gov) using the TCGAbiolinks R package (16). The overall survival (OS) information was retrieved from the TCGA Pan-Cancer Clinical Data Resource (17). The details of clinical information are shown in **Table 1A**. The patients were divided into two groups by the median expression level of GJB2 (the low GJB2 expression group and high GJB2 expression group). In addition, the normalized data of GSE75132 were downloaded from the GEO database, which included 41 cervical samples of normal morphology (30 samples with persistent HPV16 infection and 11 HPV-negative samples) (18).

**Table 1:** The clinical information of cervical cancer patients analyzed in this study.

Clinical characteristics	Subgroup	Frequency	Percentage
**a. The TCGA database**			
Total		304	
Age [Range: 20-88 (Average: 48.2, Median: 46)]	< 45	132	43.4
	≥ 45	172	56.6
Histology	Squamous cell carcinoma	252	89.0
	Adenocarcinomas	31	11.0
HPV subtype	16	103	69.1
	18	27	18.1
	45	10	6.7
	Negative	9	6.1
*FIGO	I-IIA2	189	63.4
	IIB-IV	109	36.6
Grade	G1+G2	153	56.1
	G3+G4	119	43.9
Tumor size	T1+T2	211	87.6
	T3+T4	30	12.4
Lymph node	N0	133	68.9
	N1	60	31.3
Lymphovascular invasion	NO	71	47.3
	YES	79	52.7
Distant_metastasis	NO	273	89.8
	YES	31	10.2
Vital status	Alive	233	76.6
	Dead	71	23.4
**b. The OUTDO BIOTECH Co.,LTD.**			
Total		111	
Age [Range: 29-70 (Average: 46.9, Median: 45)]	< 45	49	44.1
	≥ 45	62	55.9
FIGO	I	64	57.7
	II	25	22.5
	III	21	18.9
	IV	1	0.9
Grade	G1+G2	24	21.8
	G3	86	78.2
Lymph node	N0	90	81.1
	N1	21	18.9
Vital status	Alive	83	74.8
	Dead	28	25.2

*FIGO, Five samples descripted as FIGO stage II, which was classified as ≤ IIA2 in this study.

FIGO, The International Federation of Gynecology and Obstetrics

p values < 0.05 are highlighted as the bold values.

### Immunohistochemistry (IHC) and scoring

The tissue microarray HUteS154Su01 was obtained from Outdo Biotech. Co., Ltd. (OBC; Shanghai, China), which originally included 154 points (119 CC samples and 35 paracancerous samples). The paracancerous samples were used to verify the differential expression of GJB2. After excluding eight unqualified points (blank points, incomplete points, and points without carcinoma tissue), a total of 111 CC samples were included for survival analysis in this study. The details of clinical information are shown in **Table 1B**.

The slide was baked at 60°C for 30 mins, and then deparaffinized in xylene and passed through graded alcohol followed by antigen retrieval with 1 mM EDTA, pH 9.0 (Servicebio, G1203, Wuhan, China) in a microwave at 50°C for 10 mins and 30°C for 10 mins. The slide was incubated in 3% H_2_O_2_ for 30 mins and then washed in phosphate-buffered saline (PBS, pH 7.4) thrice. Bovine serum albumin (3%; Servicebio, G5001, Wuhan, China) was added onto the slide to cover the tissue evenly and they were subsequently incubated for 30 mins at 37°C. The slide was next incubated with the diluted antibody (ThermoFisher, 51-2800, US, dilution 1:100) overnight at 4°C. After rinsing with PBS, the slide was incubated with horseradish peroxidase-conjugated mouse antibody (Servicebio, G1214, Wuhan, China) for 50 mins, followed by diaminobenzidine (Servicebio, G1211, Wuhan, China) to detect staining under the microscope. Finally, the slide was counterstained with hematoxylin, dehydrated, and covered.

Two pathologists independently evaluated the IHC scores in a blinded manner. The stain intensity was grouped as 0 (no staining), 1 (weak staining), 2 (moderate staining), and 3 (strong staining). The percentage of each category was estimated (0%–100%). Semiquantitative histologic score (Hscore) was calculated by multiplying intensity of staining and percentage staining [Hscore = 1× (%cells 1+) + 2× (%cells 2+) + 3× (%cells 3+)] (range 0–300). Hscore ≥ 100 was considered as high expression of GJB2 in this study.

### Survival analysis

The survival, survminer, and forestplot R packages were used for survival analysis and visualization, and Kaplan-Meier survival curves were obtained for both the TCGA and OBC cohort. In addition, we explored the prognostic value of GJB2 in squamous cell carcinoma (SCC) patients, younger patients (<45), older patients (≥45), early-stage patients (FIGO stage ≤ IIA2), and late-stage patients (FIGO stage ≥ IIB). A Cox proportional hazard regression model was used for univariate and multivariate survival analysis. In univariate analysis, we evaluated the prognostic value of clinical factors and GJB2. The factors with a p value < 0.05 were used for multivariate Cox analysis to determine the prognostic value of GJB2 with clinical factors.

### Exploration of the GJB2 function

Gene expression enrichment analysis (GSEA, v.4.1.0) was used to explore the pathways related to GJB2 (19). GSEA was performed between the low GJB2 and high GJB2 expression groups. The annotated gene set c2.cp.kegg.v7.5.symbols.gmt was selected as the reference gene set. Gene set permutations were performed 1000 times for analysis. Pathways with normalized enrichment score (NES) >1, nominal p value < 0.05 and false discovery rate (FDR) q-value < 0.25 were considered as significant. To further explore GJB2 function, we obtained the top 500 genes positively correlated with GJB2 expression using the Spearman rank correlation test and used them for Gene Ontology (GO) analysis with the clusterProfiler R package (20).

### Immune microenvironment analysis

CIBERSORTx (https://cibersortx.stanford.edu) was used to analyze the effect of GJB2 expression on immune cells (21). CIBERSORTx is a deconvolution algorithm that uses a set of reference gene expression values (including 547 genes) as a minimal representation of each cell type and infers the cellular composition based on the gene expression data from bulk tumor samples with support vector regression (21). The 304 RNA-seq data were uploaded to CIBERSORTx as a mixture file, and CIBERSORTx was run with the following options: LM22 (22 immune cell types), LM22 merged into 10 major cell subsets, and disable quantile normalization. The estimate R package was used to evaluate the StromalScore, ImmuneScore, and ESTIMATEScore (22).

### Chemotherapy drugs sensitivity analysis

The natural log of the half-maximal inhibitory concentration (LN_IC50 value) of chemotherapy drugs and gene expression lists were downloaded from the Genomics of Drug Sensitivity in Cancer (GDSC), and the GDSC2 screening set was used in this study (23). We analyzed the relationship between GJB2 expression and the LN_IC50 value of cisplatin, paclitaxel, and 5-Fluorouracil, which are commonly used in CC chemotherapy.

### Statistical analysis

All statistical analyses were performed in R software (v.3.6.3), and a p value < 0.05 was considered statistically significant. The differences in GJB2 expression between the two groups were compared by t-test or Wilcoxon test. The relationship between the GJB2 expression and clinical factors was analyzed by Chi-square test and logistic regression. The survival analysis was based on the log-rank test. Spearman rank test was used to analyze the correlation.

## Results

### The GJB2 expression in cervical cancer

**Figure 1:**
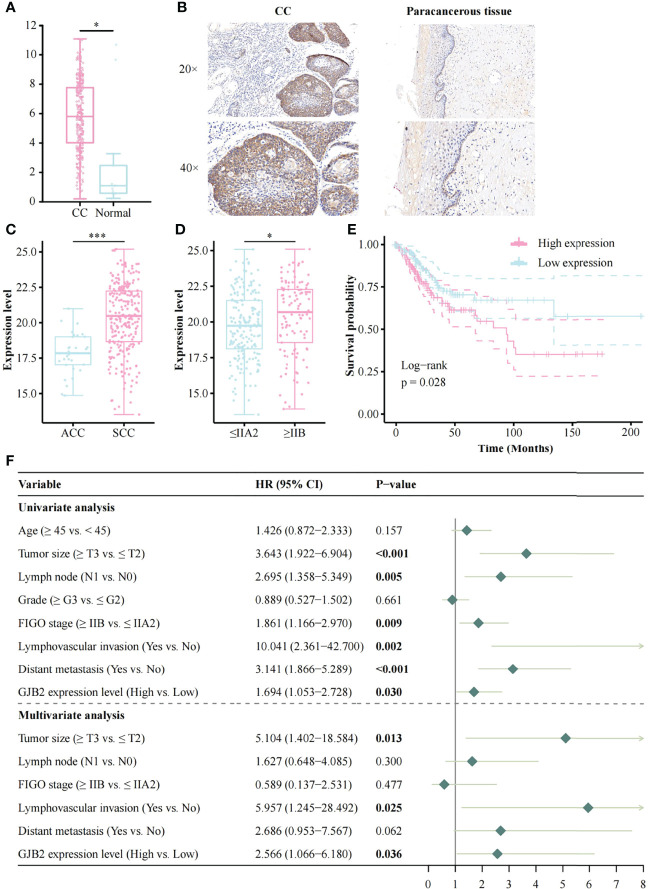
The expression of GJB2 and its association with clinical factors and survival analysis based on the TCGA database. **(A)** The result of GEPIA; **(B)** GJB2 expression in CC and its paracancerous tissue; **(C)** Histology; **(D)** FIGO stage; **(E)** Impact of GJB2 expression on overall survival in CC; **(F)** Forest plot for the univariate and multivariate Cox proportional hazard regression model. TCGA, The Cancer Genome Atlas; CC, Cervical cancer; SCC, Squamous cell carcinoma; ACC, Adenocarcinomas; FIGO, The International Federation of Gynecology and Obstetrics; HR, Hazard ratio. *p < 0.05, ***p < 0.001.

The expression of GJB2 was significantly increased in CC patients compared to normal controls (**Figure 1A**), and IHC result also indicated that GJB2 was higher in CC than in paracancerous tissue (**Figure 1B**); the result of the HPA database was consistent with the former (**Supplementary Figure 1A**). The expression of GJB2 was higher in SCC compared to adenocarcinomas (ACC, p < 0.001, **Figure 1C**). Moreover, GJB2 expression in late-stage patients (≥ IIB) was higher than that in early-stage patients (≤ IIA2) (p = 0.013, **Figure 1D**). In SCC patients, GJB2 expression level was also higher in late-stage patients (p = 0.048, **Supplementary Figure 1B**). In addition, GJB2 expression was higher in HPV16-positive samples compared to HPV-negative samples in GSE75132 (p = 0.013, **Supplementary Figure 1C**). GJB2 expression was higher in HPV-positive samples than in HPV-negative samples based on the TCGA database (p = 0.015, **Supplementary Figure 1D**). However, there was no difference in GJB2 expression among HPV16, 18, and 45 infected samples (**Supplementary Figure 1E**).

### High expression of GJB2 in CC was related to poor prognosis

**Figure 2:**
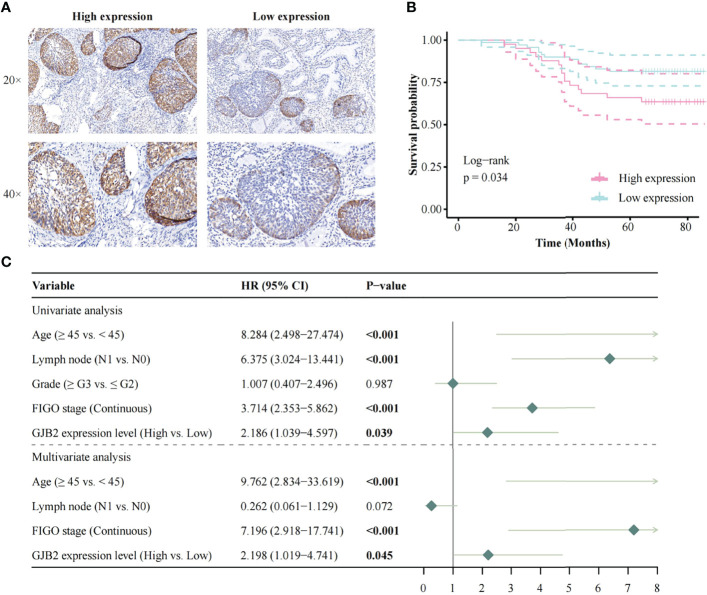
Survival analysis based on the OBC data. **(A)** The representative images of high GJB2 and low GJB2 expression; **(B)** Impact of GJB2 expression on overall survival in CC; **(C)** Forest plot for the univariate and multivariate Cox proportional hazard regression model. OBC, Outdo Biotech. Co., Ltd.; HR, Hazard ratio.

The Kaplan-Meier risk estimate was used to evaluate the relationship between GJB2 expression and the prognosis of CC patients in both TCGA and OBC cohorts. In the TCGA cohort, high GJB2 expression was significantly associated with poor OS (p = 0.028, **Figure 1E**). The median OS of patients with high GJB2 expression was 20.34 months (range: 0–176.92 months), and the median OS of patients with low GJB2 expression was 23.90 months (range: 0–210.53 months). In SCC patients, patients ≥45 years, early-stage patients (≤IIA2), and a high GJB2 expression were also related to shorter OS, and the p values were 0.019, 0.003, and 0.015, respectively (**Supplementary Figures 1F, H, I**). In the OBC cohort, the representative images of high GJB2 and low GJB2 expression are shown in **Figure 2A**. Similarly, patients with high GJB2 expression had a poorer prognosis (p = 0.034; **Figure 2B**).

### Univariate and multivariate cox analysis of the prognostic effect of GJB2

In the TCGA cohort, univariate analysis showed that tumor size (HR, 3.643; 95% CI, 1.992–6.904; p < 0.001), lymph node involvement (HR, 2.695; 95% CI, 1.358–5.349; p = 0.005), FIGO stage (HR, 1.861; 95% CI, 1.166–2.970; p = 0.009), lymphovascular invasion (HR, 10.041; 95% CI, 2.361–42.700; p = 0.002), distant metastasis (HR, 3.141; 95% CI, 1.866–5.289; p < 0.001), and high GJB2 expression (HR, 1.694; 95% CI, 1.053–2.728; p = 0.030) were correlated with patient prognosis. These factors were used for multivariate analysis and 135 patients were involved. The result indicated that tumor size (HR, 5.104; 95% CI, 1.402–18.584; p = 0.013), lymphovascular invasion (HR, 5.957; 95% CI, 1.245–28.492; p = 0.025), and high GJB2 expression (HR, 2.566; 95% CI, 1.066–6.180; p = 0.036) were independent predictors for patient prognosis (**Figure 1F**).

In the OBC cohort, the univariate analysis showed that age (HR, 8.284; 95% CI, 2.498–27.474; p < 0.001), lymph node involvement (HR, 6.375; 95% CI, 3.024–13.441; p < 0.001), FIGO stage (HR, 3.714; 95% CI, 2.353–5.862; p < 0.001), and high GJB2 expression (HR, 2.186; 95% CI, 1.039–4.597; p = 0.039) were correlated with patient prognosis. Multivariate analysis showed that age (HR, 9.762; 95% CI, 2.834–33.619; p < 0.001), FIGO stage (HR, 7.196; 95% CI, 2.918–17.741; p < 0.001), and high GJB2 expression (HR, 2.198; 95% CI, 1.019–4.741; p = 0.045) were independent predictors for patient prognosis (**Figure 2C**).

### Relationship between GJB2 and clinical factors based on the TCGA database

The patients were divided into two groups by the median expression value of GJB2. GJB2 was related to multiple clinical factors. Using the Chi-squared test, GJB2 was significantly correlated with age (p = 0.015), histology (p < 0.001), tumor size (p = 0.048), FIGO stage (p = 0.023), and differentiation grade (p = 0.001) (**Table 2**). Multiple logistic regression analysis showed that the increased expression of GJB2 was also significantly correlated with age (OR, 0.552; 95% CI, 0.349–0.874; p = 0.011), histology (OR, 20.635; 95% CI, 4.818–88.380; p < 0.001), tumor size (OR, 2.396; 95% CI, 1.070–5.364; p = 0.034), FIGO stage (OR, 1.790; 95% CI, 1.109–2.888; p = 0.017), and differentiation grade (OR, 0.429; 95% CI, 0.262–0.702; p = 0.001) (**Table 3**).

**Table 2:** Relationships between GJB2 expression and clinical factors in cervical cancer.

Clinicopathological parameters	GJB2 expression		Total	p value
	High (n=152)	Low (n=152)		
**Age**				
< 45	77	55	132	**0.015**
≥ 45	75	97	172
**Histology^#^**				
ACC	2	29	31	**<0.001**
SCC	148	104	252
**Tumor size**				
≤ T2	96	115	211	**0.048**
≥ T3	20	10	30
**Lymph node**				
N0	58	75	133	0.377
N1	31	29	60
**FIGO stage**				
≤ IIA2	85	103	188	**0.023**
≥ IIB	65	44	109
**Differentiation grade**				
≤ G2	87	66	153	**0.001**
≥ G3	43	76	119
**Lymphovascular invasion**				
No	32	39	71	1.000
Yes	36	43	79
**Distant metastasis**				
No	141	132	273	0.088
Yes	11	20	31

^#^Fisher’s exact test.

ACC, Adenocarcinomas; SCC, Squamous cell carcinoma.

p values < 0.05 are highlighted as the bold values.

**Table 3:** GJB2 expression correlated with clinical factors (logistic regression).

Clinicopathological parameters	Total (N)	OR	95% Confidence interval	p value
**Age**				
(45 vs. < 45)	304	0.552	0.349 - 0.874	**0.011**
**Histology**				
SCC vs. ACC	283	20.635	4.818 - 88.380	**<0.001**
**Tumor size**				
≥ T3 vs. ≤ T2	241	2.396	1.070 - 5.364	**0.034**
**Lymph node**				
N1 vs. N0	193	1.382	0.750 - 2.548	0.299
**FIGO stage**				
≥ IIB vs. ≤ IIA2	297	1.790	1.109 - 2.888	**0.017**
**Differentiation grade**				
≥ G3 vs. ≤ G2	272	0.429	0.262 - 0.702	**0.001**
**Lymphovascular invasion**				
Yes vs. No	150	1.020	0.536 - 1.943	0.951
**Distant metastasis**				
Yes vs. No	304	0.515	0.238 - 1.115	0.092

ACC, Adenocarcinomas; SCC, Squamous cell carcinoma.

p values < 0.05 are highlighted as the bold values.

### Exploration of GJB2-related pathways

**Figure 3:**
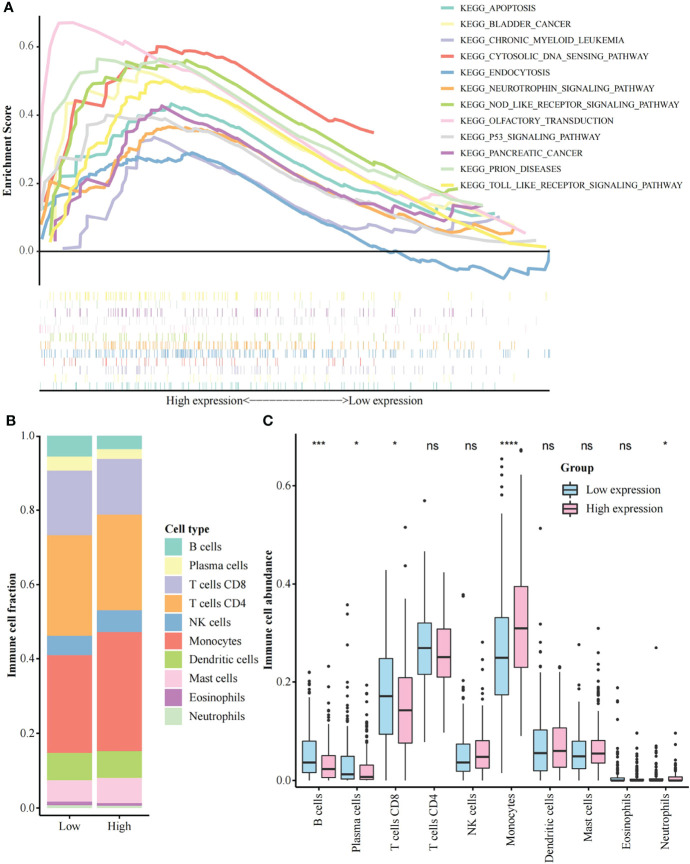
Gene set enrichment analysis and immune cell abundance analysis. **(A)** A merged enrichment plot including the enrichment score and gene sets. 12 pathways are shown here; **(B)** Proportion of immune cells in the low and high GJB2 expression group; **(C)** Immune cell abundance analysis between the low and high GJB2 expression group. *p < 0.05, ***p < 0.001, ****p < 0.0001. ns: not significant.

The GJB2 related signaling pathways were identified by GSEA. According to the selection criteria, a total of 12 pathways were significant, including apoptosis, p53 signaling pathway, and toll-like receptor (TLR) signaling pathway (**Figure 3A**, **Supplementary Table 1**). The top 500 genes positively associated with GJB2 expression were subjected to GO analysis, demonstrating that GJB2 was closely associated with epidermis structuration (**Supplementary Figure 2**, **Supplementary Table 2**).

### Effect of GJB2 expression on the immune microenvironment

CIBERSORTx was used to analyze the proportion of immune cells between the low and the high GJB2 expression group (**Figure 3B**). The abundance of B cells, plasma cells, and CD8+ T cells was significantly higher in the low GJB2 expression group than the high GJB2 expression group. Furthermore, the abundance of monocytes and neutrophils was significantly increased in the high GJB2 expression group (**Figure 3C**, **Supplementary Table 3**). In addition, according to the 22 types of cell classification, we found that the abundance of macrophages (Macrophage M0, M1, M2) was elevated in the high GJB2 expression group (**Supplementary Figure 3A**). We also found that the high GJB2 expression group had the higher ImmuneScore and ESTIMATEScore (**Supplementary Figure 3B**, **Supplementary Table 4**).

### The relationship between GJB2 expression and LN_IC50 values of cisplatin, paclitaxel, and 5-fluorouracil based on the GDSC database

**Figure 4:**
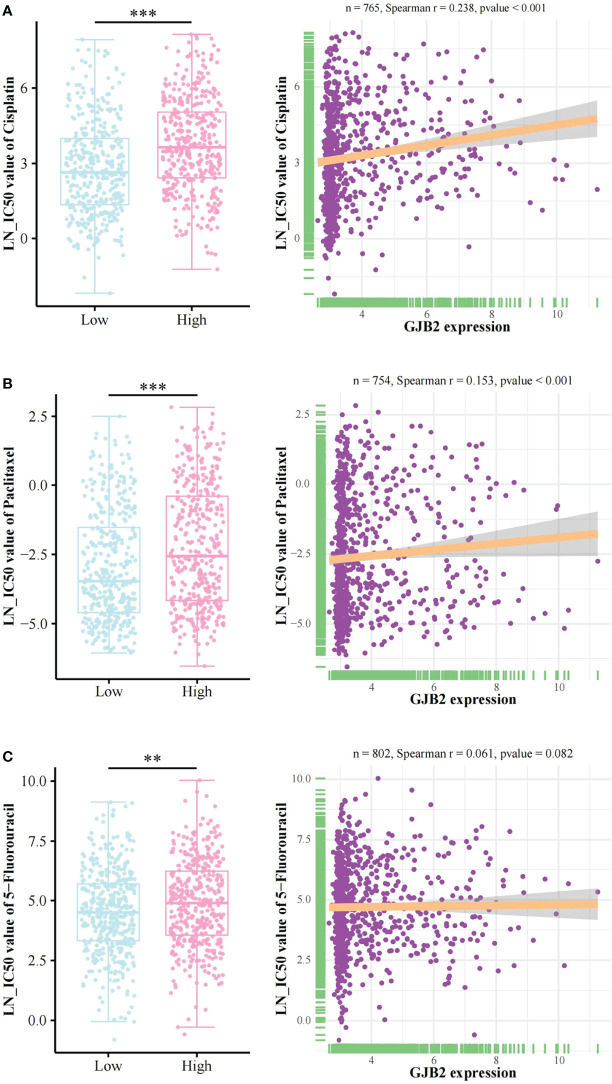
Chemotherapy drugs sensitivity analysis. The relationship between GJB2 expression and LN_IC50 values of **(A)** cisplatin, **(B)** paclitaxel, and **(C)** 5-fluorouracil. LN_IC50 value: Natural log of the half-maximal inhibitory concentration. **p < 0.01, ***p < 0.001.

The information regarding LN_IC50 values and GJB2 expression are shown in **Supplementary Table 5**. The LN_IC50 values of cisplatin, paclitaxel, and 5-Fluorouracil in the high GJB2 expression group were significantly greater than those in the low GJB2 expression group (**Figures 4A–C**). We found a positive correlation between GJB2 expression and LN_IC50 values of cisplatin/paclitaxel (Spearman r = 0.238/0.153, p < 0.001) (**Figures 4A, B**). There was no significant correlation between the GJB2 expression and LN_IC50 value of 5-fluorouracil (Spearman r = 0.061, p = 0.082) (**Figure 4C**).

## Discussion

Many studies have explored the role of GJB2 in tumors, including breast cancer, liver cancer, and colorectal cancer (10, 24, 25). However, there is a lack of research regarding GJB2 in CC. The main objective of this study was to investigate the prognostic value and function of GJB2 in CC.

We first compared GJB2 expression between CC patients and normal controls though GEPIA, the HPA database, and IHC, showing that the expression of GJB2 was higher in CC than in normal controls. Abnormal expression of GJB2 is common in tumors. Tang et al. reported that GJB2 expression was significantly increased in lung adenocarcinoma compared to normal tissue (26). Using IHC, Sun et al. verified that GJB2 expression was higher in pancreatic cancer than in para-cancerous tissue (27). In both GSE75132 and TCGA datasets, we found that GJB2 expression was higher in HPV-positive samples than in HPV-negative samples. However, GJB2 expression did not differ in HPV16, 18, and 45 infected samples. Lucke et al. reported that GJB2 was prominent in viral warts, while it was absent from normal hair-bearing skin (28). In head and neck squamous carcinoma, Méndez-Matías et al. identified that GJB2 expression was higher in HPV- patients than in HPV+ patients using the TCGA database, which is contrary to our result (29). Silva et al. found that GJB2 could co-localized with bovine papillomavirus E5 oncoprotein, suggesting a possible correlation between E5 expression and GJB2 dysregulation, and this indicates that HPV E5 oncoprotein may have a similar function (30). The relationship between GJB2 and HPV deserves further investigation.

It was observed that CC patients with high mRNA expression of GJB2 had a shorter OS (TCGA cohort) and this was validated at the protein level (OBC cohort). Additionally, GJB2 is an independent risk factor for OS by multivariate cox analysis in both cohorts. Increased expression of GJB2 was associated with poor prognosis in various tumors, including breast cancer, pancreatic cancer, lung adenocarcinoma, and esophageal squamous cell carcinoma (10, 31–33). We found that GJB2 was associated with various clinical factors in CC, such as age, tumor size, and differentiation grade. Ezumi et al. found that high expression of GJB2 was associated with venous invasion and lung metastasis in colorectal cancer (11). Tang et al. noted that the expression of GJB2 was significantly correlated with patient clinical stage, T-classification, and N-classification in lung adenocarcinoma (26).

GSEA and GO analysis were performed to investigate the functions of GJB2 in CC. A total of 12 pathways were enriched in the high GJB2 expression group, including many cancer-related pathways, such as the p53 signaling pathway and TLR signaling pathway. The proliferation, migration, and invasion of CC cells can be modulated by the p53 signaling pathway (34). TLRs are pathogenic pattern recognition receptors involved in the defense against infection and are widely expressed in a variety of tumors (35). Li et al. revealed that the TLR signaling pathway might be involved in the pathogenesis of CC (36).

We found significant differences in B cells, plasma cells, CD8+ T cells, monocytes, and neutrophils between the high and low GJB2 expression groups. In addition, we found that the expressions of macrophage M0, M1, and M2 were all predominantly increased in the high GJB2 expression group. Macrophages are the main contributor to the tumor immune microenvironment (TME), and are called tumor-associated macrophages (TAMs). TAMs can promote tumor growth and angiogenesis, reshape tissue, and inhibit acquired immunity (37). Macrophage M1 can activate the immune response and inhibit the occurrence of CC (37). Phenotype transition toward M2 correlates with poor response to chemoradiation and poor prognosis in CC (38). Macrophage M2 can increase the expression level of CD163, which can predict the malignant transformation and metastatic potential for CC (39). In addition, we calculated the StromalScore, ImmuneScore, and ESTIMATEScore. The high GJB2 expression group had higher ImmuneScore and ESTIMATEScore, which indicated that this group had more immune infiltration.

Cisplatin, paclitaxel, and 5-fluorouracil are the most commonly used drugs for CC chemotherapy (40). Thus, we analyzed the relationship between GJB2 expression and the LN_IC50 values of cisplatin, paclitaxel, and 5-fluorouracil based on the CDSC database. The lower the LN_IC50 value, the better the sensitivity to the drug. We found that the LN_IC50 values of the three drugs were significantly higher in the high GJB2 expression group than the low GJB2 expression group, indicating that GJB2 may be associated with chemoresistance in CC. The connexins family has been reported to serve as targets against chemoresistance (9). Lin et al. demonstrated that GJB4 (connexin 30.3) could promote tumor growth and induce chemoresistance *via* activation of Src (41).

There were some limitations in our study. The mechanism of how GJB2 promotes the progression of CC and affects immune cells, and the mechanism of GJB2-induced chemoresistance deserve to be further investigated in the future. In summary, overexpression of GJB2 can serve as a prognostic molecular marker of poor survival and a therapeutic target in CC. GJB2 is associated with age, tumor size, and differentiation grade in CC, and may regulate the p53 signaling pathway and TLR signaling pathway. Moreover, GJB2 can affect the TME and is correlated with chemoresistance.

## Data availability statement

Publicly available datasets were analyzed in this study. These can be found in The Cancer Genome Atlas (https://portal.gdc.cancer.gov/); the Genomics of Drug Sensitivity in Cancer (https://www.cancerrxgene.org/); the Gene Expression Omnibus (https://www.ncbi.nlm.nih.gov/geo/).

## Ethics statement

The studies involving human participants were reviewed and approved by Shanghai Outdo biotech Co. Ltd. Ethics Committee. The patients/participants provided their written informed consent to participate in this study.

## Author contributions

SM designed the overall study, analyzed the data, and performed the immunohistochemistry experiment. SM and YL prepared the figures and tables, and wrote the draft of the paper. XyW reviewed the immunohistochemistry result. XW, WX, XK, XL, and LG reviewed the statistical results. CW supervised this study. All authors contributed to the article and approved the submitted version.

## Funding

This work was supported by the National Natural Science Foundation of China (Grant number: 81974411 and 81802612).

## Acknowlegdments

The tissue microarray was obtained from Outdo Biotech. Co., Ltd. (Shanghai, China; Product Code: HUteS154Su01).

## Conflict of interest

The authors declare that the research was conducted in the absence of any commercial or financial relationships that could be construed as a potential conflict of interest.

## Publisher’s note

All claims expressed in this article are solely those of the authors and do not necessarily represent those of their affiliated organizations, or those of the publisher, the editors and the reviewers. Any product that may be evaluated in this article, or claim that may be made by its manufacturer, is not guaranteed or endorsed by the publisher.

## Supplementary material

Supplementary Figure 1 Stratified analysis of overall survival for CC patients. **(A)** GJB2 expression in the HPA database; **(B)** FIGO stage of SCC patients; **(C)** GJB2 expression between HPV-negative and HPV16-positive samples in GSE75132; **(D)** GJB2 expression between HPV-negative and HPV positive samples in TCGA database; **(E)** GJB2 expression among different type HPV-infected samples in TCGA database; **(F)** Impact of GJB2 expression on overall survival in SCC patients; **(G)** Impact of GJB2 expression on overall survival in patients < 45 years old; **(H)** Impact of GJB2 expression on overall survival in patients ≥ 45 years old; **(I)** Impact of GJB2 expression on overall survival in patients with FIGO stage ≤ IIA2; **(J)** Impact of GJB2 expression on overall survival in patients with FIGO stage ≥ IIB. HPA: The Human Protein Atlas; FIGO: The International Federation of Gynecology and Obstetrics; SCC: Squamous cell carcinoma. *p < 0.05, ns: not significant.

Supplementary Figure 2 Gene ontology analysis of the top 500 genes co-expressed with GJB2.

Supplementary Figure 3 Supplementary content for tumor immune microenvironment analysis. **(A)** Immune cell abundance analysis with 22 types of cell by CIBERSORTx; **(B)** The calculation of StromalScore, ImmuneScore, and ESTIMATEscore in the low and high GJB2 expression group. *p < 0.05, **p < 0.01, ns: not significant.

## References

[1] Sung H Ferlay J Siegel RL Laversanne M Soerjomataram I Jemal A. Global cancer statistics 2020: GLOBOCAN estimates of incidence and mortality worldwide for 36 cancers in 185 countries. CA Cancer J Clin (2021) 71(3):209–49. doi: 10.3322/caac.21660 33538338

[2] Bray F Carstensen B Møller H Zappa M Zakelj MP Lawrence G. Incidence trends of adenocarcinoma of the cervix in 13 European countries. Cancer Epidemiol Biomarkers Prev (2005) 14(9):2191–9. doi: 10.1158/1055-9965.EPI-05-0231 16172231

[3] Bray F Loos AH McCarron P Weiderpass E Arbyn M Møller H. Trends in cervical squamous cell carcinoma incidence in 13 European countries: changing risk and the effects of screening. Cancer Epidemiol Biomarkers Prev (2005) 14(3):677–86. doi: 10.1158/1055-9965.EPI-04-0569 15767349

[4] Utada M Chernyavskiy P Lee WJ Franceschi S Sauvaget C de Gonzalez AB. Increasing risk of uterine cervical cancer among young Japanese women: Comparison of incidence trends in Japan, south Korea and Japanese-americans between 1985 and 2012. Int J Cancer (2019) 144(9):2144–52. doi: 10.1002/ijc.32014 PMC7478999 30474210

[5] Bray F Lortet-Tieulent J Znaor A Brotons M Poljak M Arbyn M. Patterns and trends in human papillomavirus-related diseases in central and Eastern Europe and central Asia. Vaccine (2013) 31Suppl 7:H32–45. doi: 10.1016/j.vaccine.2013.02.071 24332296

[6] Ginsburg O Bray F Coleman MP Vanderpuye V Eniu A Kotha SR. The global burden of women's cancers: a grand challenge in global health. Lancet (2017) 389(10071):847–60. doi: 10.1016/S0140-6736(16)31392-7 PMC6191029 27814965

[7] Quinn MA Benedet JL Odicino F Maisonneuve P Beller U Creasman WT. Carcinoma of the cervix uteri. FIGO 26th annual report on the results of treatment in gynecological cancer. Int J Gynaecol Obstet (2006) 95 Suppl 1:S43–103. doi: 10.1016/S0020-7292(06)60030-1 17161167

[8] Chao X Song X Wu H You Y Wu M Li L. Selection of treatment regimens for recurrent cervical cancer. Front Oncol (2021) 11:618485. doi: 10.3389/fonc.2021.618485 33604304 PMC7884815

[9] Wu JI Wang LH. Emerging roles of gap junction proteins connexins in cancer metastasis, chemoresistance and clinical application. J Biomed Sci (2019) 26(1):8. doi: 10.1186/s12929-019-0497-x 30642339 PMC6332853

[10] Naoi Y Miyoshi Y Taguchi T Kim SJ Arai T Tamaki Y. Connexin26 expression is associated with lymphatic vessel invasion and poor prognosis in human breast cancer. Breast Cancer Res Treat (2007) 106(1):11–7. doi: 10.1007/s10549-006-9465-8 17203385

[11] Ezumi K Yamamoto H Murata K Higashiyama M Damdinsuren B Nakamura Y. Aberrant expression of connexin 26 is associated with lung metastasis of colorectal cancer. Clin Cancer Res (2008) 14(3):677–84. doi: 10.1158/1078-0432.CCR-07-1184 18245526

[12] Yang J Qin G Luo M Chen J Zhang Q Li L. Reciprocal positive regulation between Cx26 and PI3K/Akt pathway confers acquired gefitinib resistance in NSCLC cells *via* GJIC-independent induction of EMT. Cell Death Dis (2015) 6(7):e1829. doi: 10.1038/cddis.2015.197 26203858 PMC4650742

[13] Teleki I Krenacs T Szasz MA Kulka J Wichmann B Leo C. The potential prognostic value of connexin 26 and 46 expression in neoadjuvant-treated breast cancer. BMC Cancer (2013) 13:50. doi: 10.1186/1471-2407-13-50 23374644 PMC3583680

[14] Polusani SR Kalmykov EA Chandrasekhar A Zucker SN Nicholson BJ. Cell coupling mediated by connexin 26 selectively contributes to reduced adhesivity and increased migration. J Cell Sci (2016) 129(23):4399–410. doi: 10.1242/jcs.185017 PMC5201008 27777264

[15] Tang Z Li C Kang B Gao G Li C Zhang Z. GEPIA: a web server for cancer and normal gene expression profiling and interactive analyses. Nucleic Acids Res (2017) 45(W1):W98–W102. doi: 10.1093/nar/gkx247 28407145 PMC5570223

[16] Colaprico A Silva TC Olsen C Garofano L Cava C Garolini D. TCGAbiolinks: an R/Bioconductor package for integrative analysis of TCGA data. Nucleic Acids Res (2016) 44(8):e71. doi: 10.1093/nar/gkv1507 26704973 PMC4856967

[17] Liu J Lichtenberg T Hoadley KA Poisson LM Lazar AJ Cherniack AD. An integrated TCGA pan-cancer clinical data resource to drive high-quality survival outcome analytics. Cell (2018) 173(2):400–16.e11. doi: 10.1016/j.cell.2018.02.052 29625055 PMC6066282

[18] Manawapat-Klopfer A Thomsen LT Martus P Munk C Russ R Gmuender H. TMEM45A, SERPINB5 and p16INK4A transcript levels are predictive for development of high-grade cervical lesions. Am J Cancer Res (2016) 6(7):1524–36. PMC4969401 27508094

[19] Subramanian A Tamayo P Mootha VK Mukherjee S Ebert BL Gillette MA. Gene set enrichment analysis: a knowledge-based approach for interpreting genome-wide expression profiles. Proc Natl Acad Sci USA (2005) 102(43):15545–50. doi: 10.1073/pnas.0506580102 PMC1239896 16199517

[20] Yu G Wang LG Han Y He QY. clusterProfiler: an r package for comparing biological themes among gene clusters. OMICS (2012) 16(5):284–7. doi: 10.1089/omi.2011.0118 PMC3339379 22455463

[21] Newman AM Steen CB Liu CL Gentles AJ Chaudhuri AA Scherer F. Determining cell type abundance and expression from bulk tissues with digital cytometry. Nat Biotechnol (2019) 37(7):773–82. doi: 10.1038/s41587-019-0114-2 PMC6610714 31061481

[22] Yoshihara K Shahmoradgoli M Martínez E Vegesna R Kim H Torres-Garcia W. Inferring tumour purity and stromal and immune cell admixture from expression data. Nat Commun (2013) 4:2612. doi: 10.1038/ncomms3612 24113773 PMC3826632

[23] Yang W Soares J Greninger P Edelman EJ Lightfoot H Forbes S. Genomics of drug sensitivity in cancer (GDSC): a resource for therapeutic biomarker discovery in cancer cells. Nucleic Acids Res (2013) 41:D955–61. doi: 10.1093/nar/gks1111 PMC3531057 23180760

[24] Leroy K Silva Costa CJ Pieters A Dos Santos Rodrigues B Van Campenhout R Cooreman A. Expression and functionality of connexin-based channels in human liver cancer cell lines. Int J Mol Sci (2021) 22(22):12187. doi: 10.3390/ijms222212187 34830068 PMC8623148

[25] Nomura S Maeda K Noda E Inoue T Fukunaga S Nagahara H. Clinical significance of the expression of connexin26 in colorectal cancer. J Exp Clin Cancer Res (2010) 29(1):79. doi: 10.1186/1756-9966-29-79 20565955 PMC2907868

[26] Tang Y Zhang YJ Wu ZH. High GJB2 mRNA expression and its prognostic significance in lung adenocarcinoma: a study based on the TCGA database. Med (Baltimore) (2020) 99(14):e19054. doi: 10.1097/MD.0000000000019054 PMC7220691 32243356

[27] Sun D Jin H Zhang J Tan X. Integrated whole genome microarray analysis and immunohistochemical assay identifies COL11A1, GJB2 and CTRL as predictive biomarkers for pancreatic cancer. Cancer Cell Int (2018) 18:174. doi: 10.1186/s12935-018-0669-x 30410422 PMC6219000

[28] Lucke T Choudhry R Thom R Selmer IS Burden AD Hodgins MB. Upregulation of connexin 26 is a feature of keratinocyte differentiation in hyperproliferative epidermis, vaginal epithelium, and buccal epithelium. J Invest Dermatol (1999) 112(3):354–61. doi: 10.1046/j.1523-1747.1999.00512.x 10084314

[29] Méndez-Matías G Velázquez-Velázquez C Castro-Oropeza R Mantilla-Morales A Ocampo-Sandoval D Burgos-González A. Prevalence of HPV in Mexican patients with head and neck squamous carcinoma and identification of potential prognostic biomarkers. Cancers (Basel) (2021) 13(22):5602. doi: 10.3390/cancers13225602 34830760 PMC8616077

[30] Silva MA Altamura G Corteggio A Roperto F Bocaneti F Velescu E. Expression of connexin 26 and bovine papillomavirus E5 in cutaneous fibropapillomas of cattle. Vet J (2013) 195(3):337–43. doi: 10.1016/j.tvjl.2012.07.009 22892185

[31] Zhu T Gao YF Chen YX Wang ZB Yin JY Mao XY. Genome-scale analysis identifies GJB2 and ERO1LB as prognosis markers in patients with pancreatic cancer. Oncotarget (2017) 8(13):21281–9. doi: 10.18632/oncotarget.15068 PMC5400583 28177904

[32] Lu A Shi Y Liu Y Lin J Zhang H Guo Y. Integrative analyses identified ion channel genes GJB2 and SCNN1B as prognostic biomarkers and therapeutic targets for lung adenocarcinoma. Lung Cancer (2021) 158:29–39. doi: 10.1016/j.lungcan.2021.06.001 34111567

[33] Inose T Kato H Kimura H Faried A Tanaka N Sakai M. Correlation between connexin 26 expression and poor prognosis of esophageal squamous cell carcinoma. Ann Surg Oncol (2009) 16(6):1704–10. doi: 10.1245/s10434-009-0443-3 19326169

[34] Kashyap VK Dan N Chauhan N Wang Q Setua S Nagesh PKB. VERU-111 suppresses tumor growth and metastatic phenotypes of cervical cancer cells through the activation of p53 signaling pathway. Cancer Lett (2020) 470:64–74. doi: 10.1016/j.canlet.2019.11.035 31809801 PMC8059100

[35] Kumar A Yu FS. Toll-like receptors and corneal innate immunity. Curr Mol Med (2006) 6(3):327–37. doi: 10.2174/156652406776894572 PMC2666391 16712478

[36] Li J Rao H Jin C Liu J. Involvement of the toll-like Receptor/Nitric oxide signaling pathway in the pathogenesis of cervical cancer caused by high-risk human papillomavirus infection. BioMed Res Int (2017) 2017:7830262. doi: 10.1155/2017/7830262 28626766 PMC5463171

[37] Liu Y Li L Li Y Zhao X. Research progress on tumor-associated macrophages and inflammation in cervical cancer. BioMed Res Int (2020) 2020:6842963. doi: 10.1155/2020/6842963 32083131 PMC7011341

[38] Petrillo M Zannoni GF Martinelli E Pedone Anchora L Ferrandina G Tropeano G. Polarisation of tumor-associated macrophages toward M2 phenotype correlates with poor response to chemoradiation and reduced survival in patients with locally advanced cervical cancer. PloS One (2015) 10(9):e0136654. doi: 10.1371/journal.pone.0136654 26335330 PMC4559430

[39] Chen XJ Han LF Wu XG Wei WF Wu LF Yi HY. Clinical significance of CD163+ and CD68+ tumor-associated macrophages in high-risk HPV-related cervical cancer. J Cancer (2017) 8(18):3868–75. doi: 10.7150/jca.21444 PMC5688941 29151975

[40] Xu W Xie S Chen X Pan S Qian H Zhu X. Effects of quercetin on the efficacy of various chemotherapeutic drugs in cervical cancer cells. Drug Des Devel Ther (2021) 15:577–88. doi: 10.2147/DDDT.S291865 PMC7894806 33623367

[41] Lin YP Wu JI Tseng CW Chen HJ Wang LH. Gjb4 serves as a novel biomarker for lung cancer and promotes metastasis and chemoresistance *via* src activation. Oncogene (2019) 38(6):822–37. doi: 10.1038/s41388-018-0471-1 30177841

