# Supplementary figures and images for: The prognostic value and biological significance of gap junction beta protein 2 (GJB2 or Cx26) in cervical cancer
Source: Front Oncol. 2022 Jul 21;12:907960. doi: 10.3389/fonc.2022.907960 (PMC9355537; doi:10.3389/fonc.2022.907960)

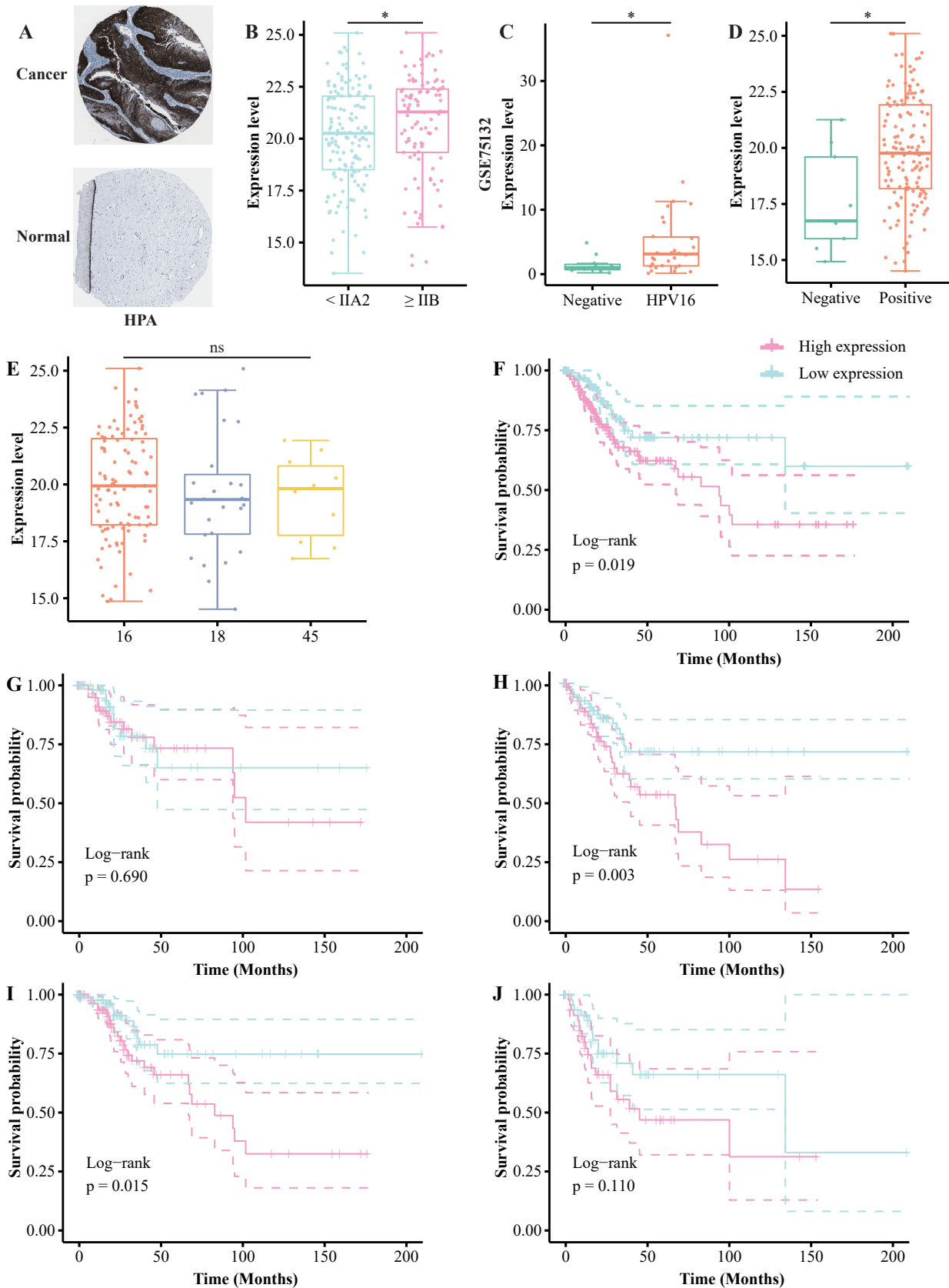

Supplement: Supplementary Figure 1 — Stratified analysis of overall survival for CC patients. (A) GJB2 expression in the HPA database; (B) FIGO stage of SCC patients; (C) GJB2 expression between HPV-negative and HPV16-positive samples in GSE75132; (D) GJB2 expression between HPV-negative and HPV positive samples in TCGA database; (E) GJB2 expression among different type HPV-infected samples in TCGA database; (F) Impact of GJB2 expression on overall survival in SCC patients; (G) Impact of GJB2 expression on overall survival in patients < 45 years old; (H) Impact of GJB2 expression on overall survival in patients ≥ 45 years old; (I) Impact of GJB2 expression on overall survival in patients with FIGO stage ≤ IIA2; (J) Impact of GJB2 expression on overall survival in patients with FIGO stage ≥ IIB. HPA: The Human Protein Atlas; FIGO: The International Federation of Gynecology and Obstetrics; SCC: Squamous cell carcinoma. *p < 0.05, ns: not significant. [file DataSheet_1.zip › Supplementary Figure 1.pdf]

GeneNumber

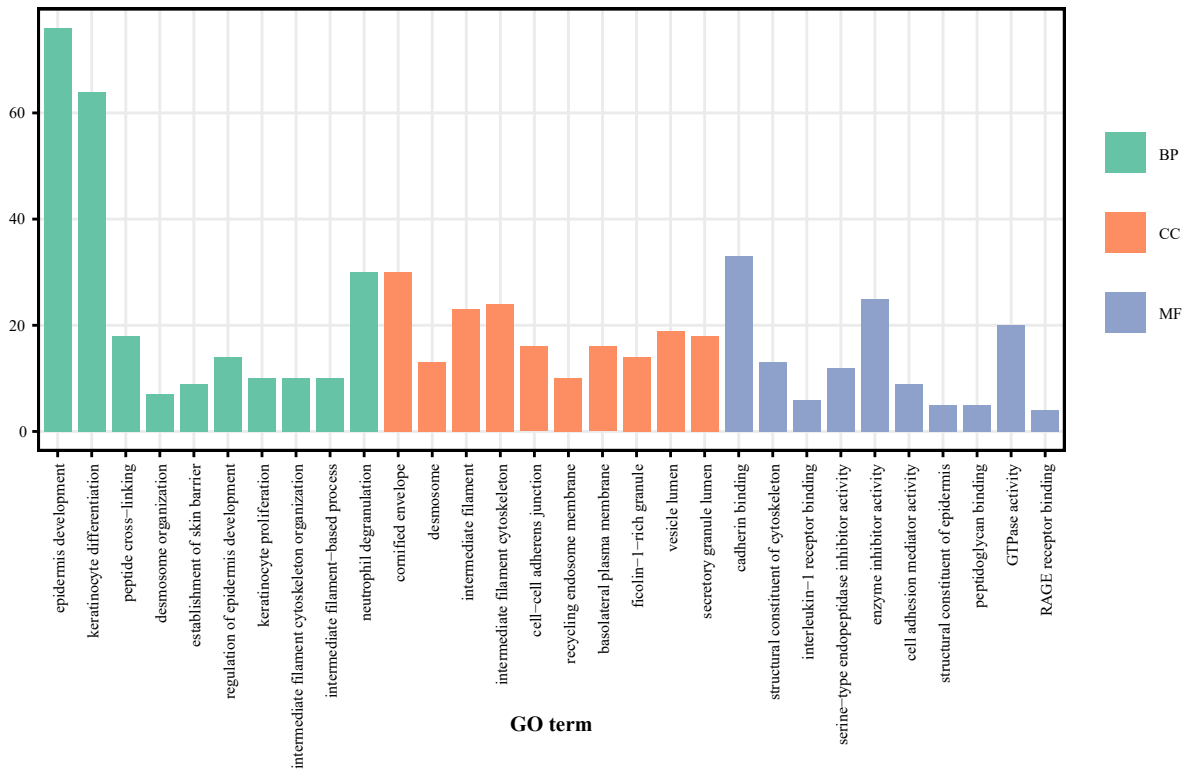

Supplement: Supplementary Figure 1 — Stratified analysis of overall survival for CC patients. (A) GJB2 expression in the HPA database; (B) FIGO stage of SCC patients; (C) GJB2 expression between HPV-negative and HPV16-positive samples in GSE75132; (D) GJB2 expression between HPV-negative and HPV positive samples in TCGA database; (E) GJB2 expression among different type HPV-infected samples in TCGA database; (F) Impact of GJB2 expression on overall survival in SCC patients; (G) Impact of GJB2 expression on overall survival in patients < 45 years old; (H) Impact of GJB2 expression on overall survival in patients ≥ 45 years old; (I) Impact of GJB2 expression on overall survival in patients with FIGO stage ≤ IIA2; (J) Impact of GJB2 expression on overall survival in patients with FIGO stage ≥ IIB. HPA: The Human Protein Atlas; FIGO: The International Federation of Gynecology and Obstetrics; SCC: Squamous cell carcinoma. *p < 0.05, ns: not significant. [file DataSheet_1.zip › Supplementary Figure 2.pdf]

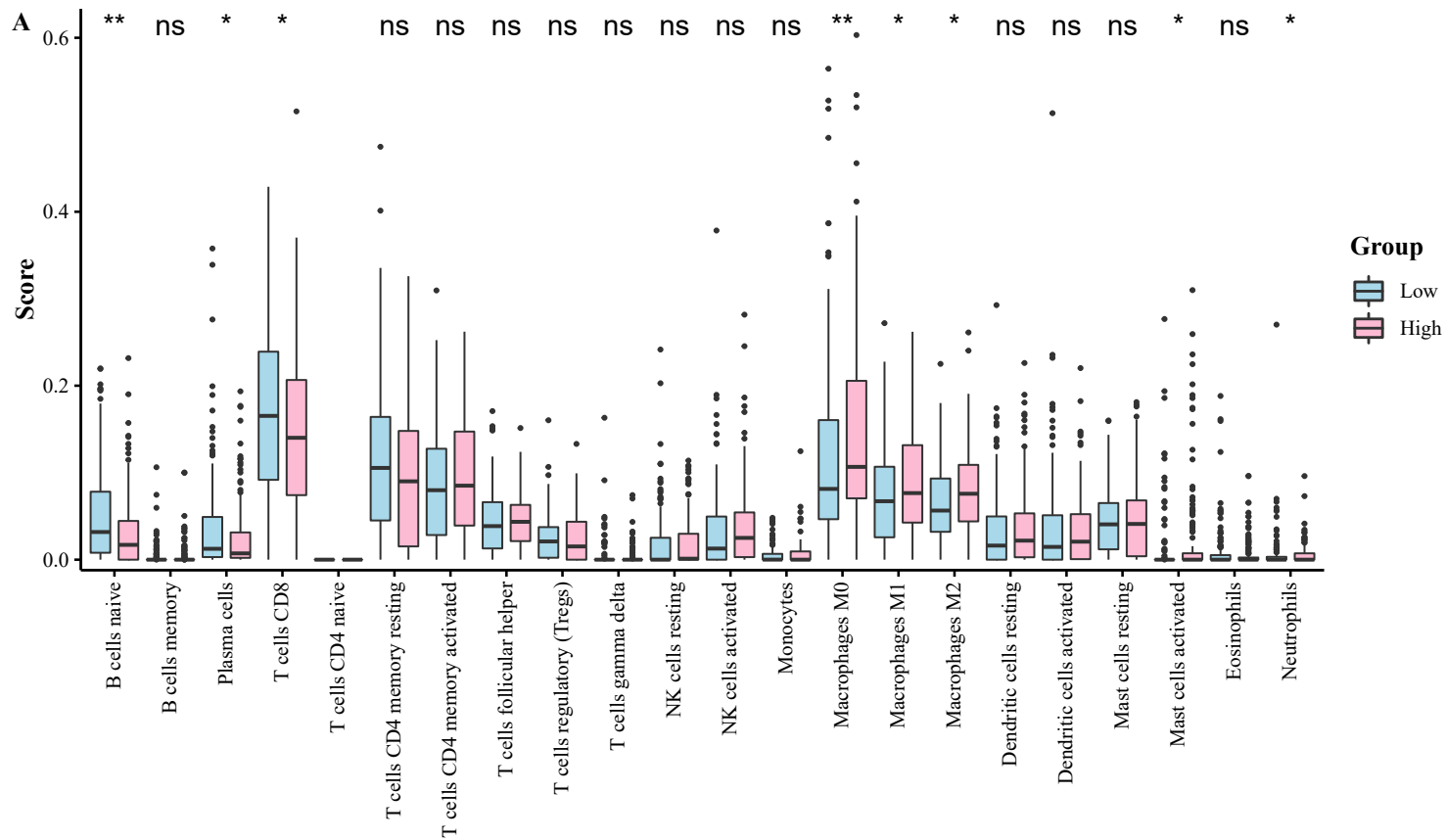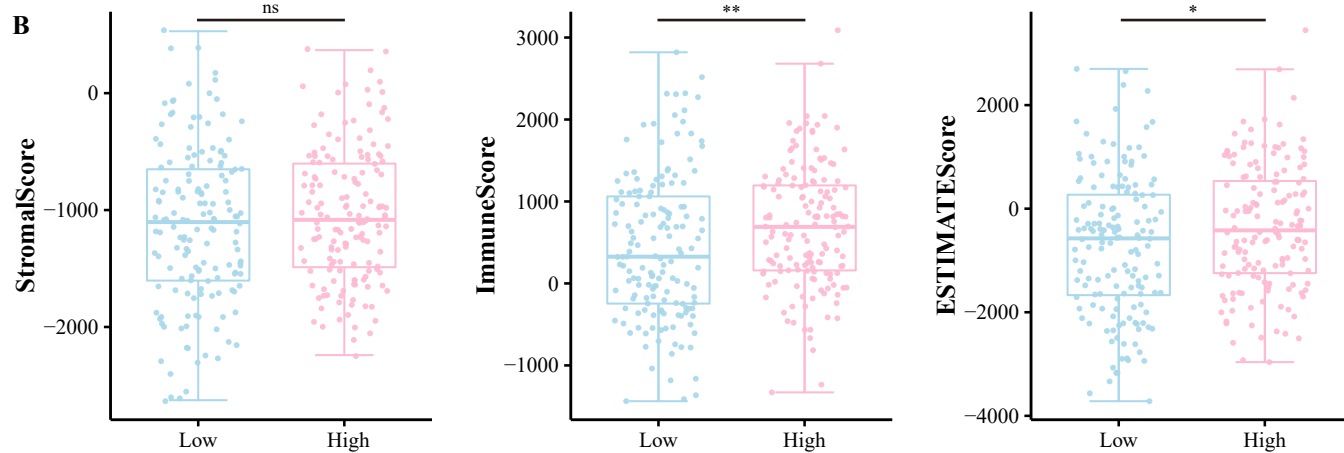

Supplement: Supplementary Figure 1 — Stratified analysis of overall survival for CC patients. (A) GJB2 expression in the HPA database; (B) FIGO stage of SCC patients; (C) GJB2 expression between HPV-negative and HPV16-positive samples in GSE75132; (D) GJB2 expression between HPV-negative and HPV positive samples in TCGA database; (E) GJB2 expression among different type HPV-infected samples in TCGA database; (F) Impact of GJB2 expression on overall survival in SCC patients; (G) Impact of GJB2 expression on overall survival in patients < 45 years old; (H) Impact of GJB2 expression on overall survival in patients ≥ 45 years old; (I) Impact of GJB2 expression on overall survival in patients with FIGO stage ≤ IIA2; (J) Impact of GJB2 expression on overall survival in patients with FIGO stage ≥ IIB. HPA: The Human Protein Atlas; FIGO: The International Federation of Gynecology and Obstetrics; SCC: Squamous cell carcinoma. *p < 0.05, ns: not significant. [file DataSheet_1.zip › Supplementary Figure 3.pdf]
